# Middle-range theory of the existential dimension of being-in-the-world of chronic kidney disease: Grounded Theory

**DOI:** 10.1590/0034-7167-2023-0152

**Published:** 2024-09-20

**Authors:** Carolina Giordani da Silva, Maria da Graça Oliveira Crossetti, Maravilla Giménez Fernández, Janaína dos Santos Prates

**Affiliations:** I Universidade Federal do Mato Grosso. Cuiabá, Mato Grosso, Brazil; II Universidade Federal do Rio Grande do Sul. Porto Alegre, Rio Grande do Sul, Brazil; III Universidad Católica San Antonio de Murcia. Murcia, Murcia, Spain

**Keywords:** Nursing Theory, Existentialism, Nursing, Grounded Theory, Chronic Kidney Disease, Teorías de Enfermería, Existencialismo, Enfermería, Teoría Fundamentada, Enfermedad Renal Crónica

## Abstract

**Objectives:**

to develop a middle-range theory of the existential dimension of being-in-the-world of chronic kidney disease in the light of humanistic nursing theory.

**Methods:**

exploratory-descriptive, qualitative, Grounded Theory study, whose guiding question was: what theoretical relationships can be established between clinical practice carried out in the context of renal replacement therapies and the concepts of humanistic nursing theory? Data were collected through semi-structured interviews with nursing staff and chronic kidney disease patients.

**Results:**

a theory was developed that describes and explains the existential dimension of being-in-the-world of chronic kidney disease, identifying the qualitative evidence present in this context.

**Final Considerations:**

the theory contributes to clinical nursing practice qualification in nephrology, consolidating nursing as an art and science, because it arises from care practice and research, in addition to rescuing what differentiates it within the health disciplines, which is care par excellence.

## RESUMEN

**Objetivos:**

desarrollar una teoría intermedia de la dimensión existencial del estar-en-el-mundo de la enfermedad renal crónica a la luz de la teoría humanista de la enfermería.

**Métodos:**

estudio exploratorio-descriptivo, cualitativo, de Teoría Basada en Datos, cuya pregunta orientadora fue: ¿qué relaciones teóricas se pueden establecer entre la práctica clínica realizada en el contexto de las terapias de reemplazo renal y los conceptos de la teoría humanista de enfermería? Los datos fueron recolectados a través de entrevistas semiestructuradas con personal de enfermería y pacientes con enfermedad renal crónica.

**Resultados:**

se desarrolló una teoría que describe y explica la dimensión existencial del estar-en-el-mundo de la enfermedad renal crónica, identificando la evidencia cualitativa presente en este contexto.

**Consideraciones Finales:**

la teoría contribuye a la calificación de la práctica clínica de enfermería en el contexto de la nefrología, consolidando la enfermería como arte y ciencia, porque nace de la práctica del cuidado y de la investigación, además de rescatar lo que lo diferencia dentro de las disciplinas de la salud, que es el cuidado por excelencia.

## INTRODUCTION

Chronic kidney disease (CKD) is characterized by abnormalities in the structure or function of the kidneys present for more than three months, in addition to the progressive and irreversible loss of kidney function, being part of the group of chronic noncommunicable diseases, which in the stage more advanced, stage 5, dialysis, represents the most severe degree of the disease, leading to the need for Renal Replacement Therapy (RRT) to maintain life^(1-2)^.

In Brazil, according to the latest census published in 2023 by the Brazilian Society of Nephrology, the estimated total number of people with CKD undergoing stage 5 dialysis reached 148,363, with an average increase of 5,468 patients (3.5%), compared to last year^(3)^. The most predominant RRT for CKD is hemodialysis, with a prevalence of up to 95.3% of patients, followed by peritoneal dialysis and kidney transplantation. RRT, although it is the only possibility of survival for patients, brings with it a series of changes inherent to the physical dimension, but also to the existential condition, related to the uncertainty of the future, the proximity to death and the fear of this new world that presents itself^(2)^.

Existential condition refers to man as a singular being, who show themselves in everyday life with forms of expression that can be authentic, unique and singular, or inauthentic, inappropriate and impersonal. From an existential perspective, human beings have the ability to see, signify and appropriate things in the world from their own perspective^(4)^.

In the context of CKD, this existential condition is related to the feelings expressed by patients, recognized as qualitative evidence in the context of care and which requires the nursing team to look at all these needs, physical and existential, to provide effective and safe quality care, minimizing the risk for patients and professionals^(5)^.

In this regard, it is believed that, through theoretical frameworks that guide nurses to identify these feelings when carrying out the Nursing Process (NP), these can be considered in care planning, through diagnoses and nursing care that reflect the individuals’ existential dimension, making them unique and complete, as human beings are seen not only in their biological dimension, but also in the existential, emotional, spiritual and social dimensions, contributing to patient adherence and acceptance to proposed treatment.

Nursing theories contribute to organization of care, as they act as a reference or organizing structure, functioning as a symbolic representation of aspects of reality to guide professional practice. They aim to describe, explain, predict or prescribe conditions or relationships of phenomena, guiding the application of NP in different contexts^(6)^.

Among the types of theory, middle-range theory (MRT) stands out, defined as a set of related ideas that are focused on a specific dimension of a phenomenon, including a restricted number of concepts and propositions, described at a specific level concrete, which are directly linked to research and practice, which generate hypotheses that can be tested through empirical research. MRT for nursing is classified, according to its purpose, into descriptive, explanatory and predictive^(7)^.

From this perspective, it is inferred that the development of a specific MRT for nursing based on a major theory^(7-8)^, such as Paterson’s and Zderad’s humanistic nursing theory^(9)^ (1979), can support nurses to identify the qualitative evidence of patients in RRT, whose existential condition needs to be assessed in the nursing care process, since the lack of understanding of the health-disease process and non-acceptance of their condition negatively impact patient treatment. It is believed that the humanistic theory^(9)^, when it concretely proposes that nurses approach nursing consciously and deliberately as an existential experience, meets this way of looking at “human beings” in their singularity, filling this gap in clinical practice and improving the quality of care provided to these patients in RRT, as this population has been growing year by year^(3)^.

Thus, the following question emerged in this study: what theoretical relationships can be established between the clinical practice carried out in the context of RRT and the concepts of the Paterson and Zderad^(9-10)^ theoretical framework for developing a MRT?

## OBJECTIVES

To describe the development of a MRT of the existential dimension of being-in-the-world of CKD based on Paterson and Zderad’s humanistic nursing theory^(9)^ framework with input from Grounded Theory (GT)^(11)^.

## METHODS

### Ethical aspects

The study was conducted in accordance with national and international ethics guidelines, and was approved firstly by the *Universidade Federal do Rio Grande do Sul* Research Ethics Committee and then by the *Hospital de Clínicas de Porto Alegre* Research Ethics Committee.

### Theoretical framework

The humanistic nursing practice theory proposes that nurses approach nursing with deliberate awareness that it is an existential experience. Thus, when reflecting on their concepts, they can phenomenologically describe the stimuli they receive, their responses and what they come to know through their presence in the situation of caring for others^(9)^.

Humanistic nursing is an experience lived between human beings; therefore, it is more than a unilateral subject-object relationship, technically competent and charitable, guided for the benefit of another. It is a transactional relationship that is responsible for investigating, whose expression demands conceptualization based on the existential awareness that nurses have of their being and of others^(9)^.

### Study design

This is an exploratory-descriptive qualitative study^(11)^, with the GT methodological framework, in Charmaz’s^(12)^ constructivist aspect, following the EQUATOR instrument - Standards for reporting qualitative research^(13)^.

### Methodological procedures

The study took place at a university hospital in Rio Grande do Sul, Brazil, in the nephrology service, which treats acute or chronic kidney patients in need of RRT.

The study population was made up of nurses and nursing technicians who work in the nephrology unit and patients who were undergoing some type of RRT. Participants were defined by convenience, through an invitation to participate in the study.

The initial sample consisted of five nurses, three nursing technicians and nine patients. However, according to theoretical sampling, in attention to the methodological framework^(12)^, as data was collected and theoretical concepts gained density, from constant comparison, use of memos and inductive-deductive thinking, hypotheses emerged, guiding the expansion of participants, resulting in a final sample composed of seven nurses, three nursing technicians and ten patients. This quantity was defined from the moment that the information from the participants did not provide relevant data towards new categorizations and formulation of new theoretical concepts.

Inclusion criteria comprised nurses and nursing technicians with at least one year of experience at the study institution, present during the data collection period, and patients who were in some type of RRT, able to answer the interview. Exclusion criteria comprised nurses and nursing technicians who were not providing assistance during the research period as well as patients who had cognitive or neurological deficits that did not allow them to respond to the survey and presented difficulties with the use of technologies, such as software applications cell phone and/or computer for virtual interviews.

Data collection and analysis took place from January 2020 to January 2021, concomitantly, emphasizing the elaboration of analysis of the action and process. In this way, simultaneous helps to pursue this emphasis as it adapts to data collection, to inform emerging analyzes^(12)^. Semi-structured interviews were carried out virtually through the Zoom application, after signing the Informed Consent Form (ICF), which was obtained from all individuals in the study by written means.

The interviews lasted between 25 and 120 minutes, and were all recorded. In consideration of the methodological framework adopted in this study^(12)^, memos were created, which are similar to field diaries, in which the researcher, who is a nurse specializing in nephrology and with solid experience in RRT, recorded his thoughts regarding the topic, comparisons and connections made, and indicated the questions and directions to be followed.

For data analysis, the NVivo 12 software resource was used to perform the coding^(12)^. In this way, three types of coding were carried out: initial, fragmenting the data and creating “in vivo” codes from the statements; focused, integrating, organizing and synthesizing data by similarity towards the categories and axial, relating the categories and specifying their properties towards the definition of concepts, assumptions, relational statements, empirical indicators and structure; and MRT contextualization of the existential dimension of CKD’s being-in-the-world, based on the assumptions and concepts of Paterson and Zderad’s humanistic nursing theory, such as being, encounter, concern, empathy, “being-more”, belonging, among others, which guided MRT construction, bringing concepts together and establishing the relationship between them^(9)^.

## RESULTS

Participants representing nursing professionals were predominantly female, with only two professionals, a nurse and a nursing technician, being male. Regarding nurses, two nurses from hemodialysis, two from peritoneal dialysis and three from kidney transplant participated. All nursing technicians worked in hemodialysis RRT, with experience ranging from five to 25 years of experience in RRT.

As for participants, there was a predominance of females, with only three male patients, aged between 26 and 66 years, with a predominance of five patients aged between 32 and 39 years, demonstrating CKD involvement in young adults. The prevalent RRT was hemodialysis, performed by four patients, followed by three peritoneal dialysis and three kidney transplant recipients. However, it is noteworthy that, even patients who at the time of the interview were undergoing another type of RRT, all at some point underwent hemodialysis.

In relation to theory, it presents several components such as the purpose, concepts, statements, conceptual structure, assumptions and propositions. It is clarified that the expression “in the world of CKD” used in this theory has a philosophical nature, referring to the environment, the physical space in which RRT occurs, and that “nursing-being” and “patient-being”, which is “being-in-the-world with CKD” coexist.

The MRT of the existential dimension of being-in-the-world of CKD in its purpose is descriptive and explanatory; therefore, it proposes to guide “nursing-being” to identify the existential dimension in the NP carried out by “being a patient” through recognition qualitative evidence present in this context, providing more humanized care, valuing the uniqueness of beings-in-the-world with CKD in the different MRT, helping them feel like they belong to this world and can thus develop their “being-more” or their greatest potential^(10)^.

Chart 1 presents the concepts and assumptions that structure the theory, based on the categories and subcategories that emerged in data analysis. It is noteworthy that the structuring elements are information contained in a determined amount of data, allowing a more comprehensive understanding of the phenomenon studied^(14)^, resulting from the coding process proposed by the methodology adopted in this study.

**Chart 1:** Components of proposed theory

Category	Subcategory	Structuring elements	Theory component
Unveiling the being-in-the-world of chronic kidney disease	Patient-being	Being in the world with CKD	Concept
		Losing autonomy due to limitations	
		Used to being taken care of	
		Transcending the CKD	
	Nursing-being	Unveiling possibilities	Concept
		Being a mediator	
		Being present	Assumption
	Meeting	Establishing links	Assumption
		Becoming a family	
	Becoming concerned	Being empathetic	Assumption
Context of care	Environment	Adapting to reality	Concept
		Establishing a living space	
	Temporality	Becoming a routine	Concept
		Passing the time	Assumption
Transcending the world of CKD	Accepting the CKD	Renouncing their possibilities	Concept
		Projecting oneself into the world with CKD	
	Designing possibilities to transcend	Establishing coping strategies	Concept
	Anxieties of being-in-the-world in CKD	Recognizing uncertainties	Assumption
	Motivating to transcend		Assumption

*CKD - chronic kidney disease.*

Below, each category is presented with its components.

### Category - Unveiling the being-in-the-world of chronic kidney disease

This category made it possible to recognize complex “patient-being” and “being-in-the-world with CKD”, with many uncertainties, limitations, in the face of a situation that generally presents itself suddenly, and “nursing-being”, made up of nurses and nursing technicians, who have autonomy, who care about “patient-being” and who, with empathy and presence, at each care encounter, seek, through knowledge and creativity, excellent care for “patient-being”, in which both can project themselves towards their “being-more”.

*Chronic kidney patients have very peculiar characteristics that range from the different underlying diseases they may have, hypertension, DM, familial diseases, such as polycystic kidneys,* [...]. *I see him as a patient with several limitations and who will be undergoing treatment for a long period of time.* (NUR2)

*I don’t have a normal life,* [...]. *We try, but we don’t take it. Leaving college, changing the way I eat, how I live, having to think more about what I’m eating, what I’m drinking. Losing my freedom.* (P9)

*After the transplant, I just thought that I was free from the machines, I wouldn’t suffer anymore.* (P8)

### Category - Context of care

It is understood as an environment that is a living, dynamic space of interrelationships between beings-in-the-world of CKD who, in the temporality of each one, share experiences, seeking to adapt to reality to better be in this world, what happens over time. In this scenario, care occurs when a space for social interaction is established that allows beings-in-the-world with CKD to seek belonging to this environment.

*I had to readapt to this new life* [...] *the bad part of peritoneal dialysis is this, you are alone, in the other, you were friends, you talked, but I didn’t have medical support. In this case, I have it, so everything has pros and cons.* (P9)

*The environment is pleasant because it is not an environment like that with absolute silence. Technicians, people play with each other, all within one volume. There is life inside the room, it is not a dead thing.* [...], *a normal life works there.* [...] *in the room* [...] *I see life, I see movement.* (P6)

*I think for some patients it is a social event. I have patients who have undergone transplantation and say what they miss is the coexistence they had on hemodialysis.* (NUR6)

### Category - Transcending the world of chronic kidney disease

Transcending the world of CKD requires “patient-being” accepting CKD with all its complexity and, therefore, when encountering “nursing-being”, together sharing their anxieties, seeking to motivate themselves to continue being and, thus, project possibilities to reach their best potential, i.e., their “being-more”.

*When I started undergoing treatment, I didn’t understand what was happening for a long time.* [...] *I didn’t really understand what a kidney problem was, so there was a lot of uncertainty* [...] *we don’t know what it’s like, it makes us anxious.* (P10)

*I think, yes, motivation is really a nurse’s role, I think within dialysis a lot too. It gives a conversation and really motivates patients to want to continue doing well; gives a compliment to patients, they already feel better, more motivated, loved and useful.* (NUR7)

*Look, girl, the transplant gave me a new life, it would improve my life for the better* [laughs]. *I just took it, I think I got ready and left. I said, “Thank God, I’m going to get off these machines, it gives me relief”.* (P8)

**Figure 1:**
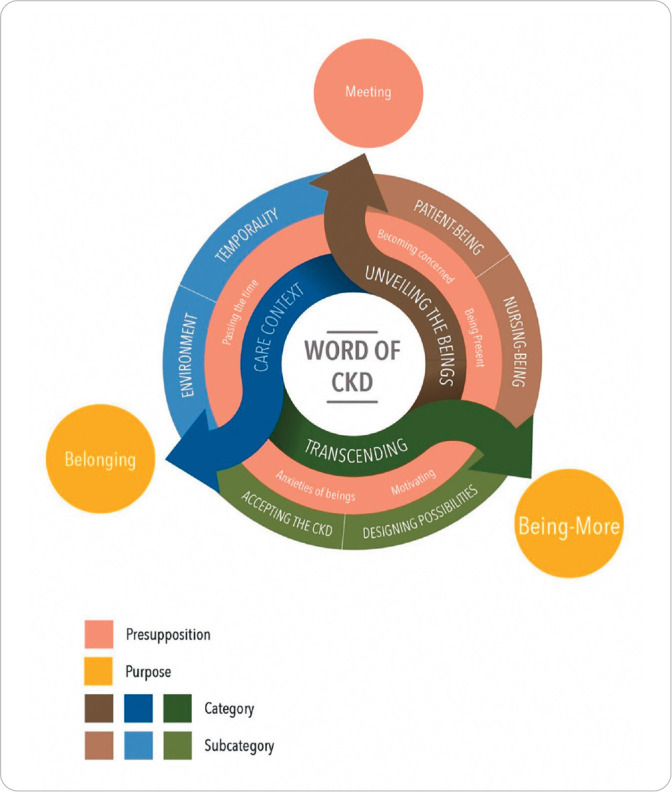
Model diagram of the existential dimension of c hronic kidney disease’s being-in-the-world

These unveiled concepts and assumptions direct CKD’s being-in-the-world to two purposes, belonging and “being-more”, as can be seen in the MRT Model diagram of the existential dimension of CKD’s being-in-the-world (Figure 1).

After identifying the main concepts, non-relational statements were created, which correspond to the theoretical or operational definition of each one, as described below:

“Patient-being” - “being-in-the-world with CKD”, complex and experiencing the anxieties of this world in the face of so many uncertainties. It presents fear, sadness, hopelessness, anger, mourning and suffering due to loss of autonomy and insecurity due to constantly living in close proximity to death. However, in relationships with other beings, they perceive possibilities to continue being, which gives them hope and joy in achieving their “more-being”^(10)^.

“Nursing-being” - “being-in-the-world of CKD” that cares through authentic presence, driven by concern in response to a call to “patient-being”. It presents many specificities, such as autonomy, specific knowledge to create possibilities in the face of the challenges of this world, creativity and empathy to perceive and establish bonds with other beings in this context^(10)^.

“Environment” - physical space, the world of CKD, the context of care that concerns not only the physical space, but, above all, the interrelationships that are built in it over time and that reflect the way in which beings continue to exist in the world. It is a type of empty, personalized space, to which one feels belonging^(10)^.

“Temporality” - is the passage of time, understanding that there is an experience of the internal time of the being, where self-awareness resides, and of external time, inherent to the things of the world, and that it is in the experience of temporality and resolved existence that care is rooted, which allows the being to develop a feeling of belonging^(10)^.

“Accepting the CKD” - a condition resulting in which “patients” understand that they are sick, but that they are not alone, that they can have a support network to help them and that there are possibilities to continue projecting themselves into the world towards their “being-more” through a RRT^(10)^.

“Projecting possibilities to transcend” - the ability of the being-in-the-world with CKD to reinvent themselves to meet the demands of their immediate surroundings and find a balance between their expectations and the situation they are experiencing^(10)^.

Assumptions are understood as preconceived beliefs and accepted as truths on which the theory is based, without requiring proof and, therefore, are not tested empirically^(8)^. The new MRT has six assumptions, which are:

“Being present” - condition for the lived dialogue of nursing to effectively occur in encounters between “patient-being” and “nursing-being”, which requires availability, with the intention of doing and being in a relationship, experiencing the shared situation^(10)^.

“Meeting” - meeting of singular beings in the world of CKD, in a movement that requires presence and openness so that they can reveal themselves to each other and, thus, coexist, establish bonds and continue towards their “being-more”^(10)^.

“Becoming concerned” - essential mode of “being with”, which moves the being towards the care encounter in which there is a call and a response, allowing a transactional relationship^(10)^.

“Passing the time” - condition of being in the world of CKD, necessary so that “patient-being” and “nursing-being”, when interacting, can reveal new possibilities to transcend and continue coexisting towards the future, singularly, in their internal rhythm, to adapt to this new reality^(10)^.

“Anxieties of beings-in-the-world of CKD” - the deepest feeling of “being”, which is the principle and source of all others. It is related to the unpredictability and uncertainties that CKD and RRT themselves cause^(10)^.

“Motivating to transcend” - meaning or reason to continue “being-being” and, thus, discover possibilities to transcend and project oneself in the world^(10)^.

Relational statements or propositions refer to some type of relationship between two or more theory concepts, necessary for its construction, and may state an association (correlation) or causality^(6)^. According to the GT method used, these propositions were made during coding towards category construction, through the creation of hypotheses that directed theoretical sampling and theory modulation. In this regard, the propositions identified in the new MRT are presented:

“Patient-being” and “nursing-being” mutually influence the care encounter, as “nursing-being” goes towards “patient-being” driven by “becoming concerned”. Hence, “meeting” depends on the willingness of “patient-being” to continue being in this world and, thus, transcend CKD, and on “nursing-being” being present in this meeting, to reveal possibilities for taking care of “patient-being”.

“Environment” influences “meeting”, as it may or may not enable a coexistence space that interferes with CKD acceptance and the feeling of belonging through “patient-being” in this world in which “temporality” will also influence, as adaptation to RRT is an individual process. Therefore, “meeting” modulates the establishment of bonds between “patient-being” and “nursing-being” towards becoming a family, in which the “passing the time” influences and also modulates these meetings, towards each “being” reaching its “being-more”.

“Becoming concerned” influences and modulates the response of “nursing-being” to “patient-being”, which seeks, through empathy and mediation, to influence “CKD acceptance” by “patient-being”, “motivating” them to, together, find “coping strategies” for CKD. In this regard, “motivating” influences and modulates “patient-being”, “nursing-being”, as well as “projecting possibilities in the world of CKD”, being a condition for “transcending the world of CKD”.

“Transcending the world of CKD” suffers interference from “anxieties of beings-in-the-world of CKD”, to the extent that this, often, makes it difficult for “patient-being” to adhere to RRT and “nursing-being” to uncover possibilities for caring for “patient-being”, which can also impact bond establishment in “meeting”^(10)^.

## DISCUSSION

Existentially, “being” is seen as a concrete and lived experience towards “becoming” through their choices. The lived experiences have man as an object of study seen as an experience, an unfinished project of work, becoming a reality to be constantly discovered^(9,15)^. Thus, it is necessary to recognize each man as a unique existence in their situation, relating to others in time and space. It is through their relationships with other men that men come to be and that their singular individuality is realized^(9,15)^.

In this sense, “patient-being”, when faced with the world of CKD, finds themselves surrounded by uncertainty, as this presents itself as a sudden, unexpected event, generating suffering^(16)^, in addition to bringing with it complications inherent to the underlying disease and CKD itself, requiring limitations that provoke feelings of revolt, fear, sadness, hopelessness and even social isolation.

Furthermore, hemodialysis and peritoneal dialysis cause “patient-being” the emotional burden of giving up their freedom while spending their days connected to a machine, which also gives rise to a feeling of loss^(17-20)^ of their autonomy, causing significant changes and suffering, a burden also mentioned by transplant patients when they underwent such therapies and which ceased to exist with transplantation. Therefore, despite this, “patient-being” is able to express gratitude for the possibility of receiving dialysis^(14,20)^, and throughout treatment, when a feeling of belonging emerges, he begins to have a perspective on the future in the hope of undergoing a transplantation.

Hence, nursing, from an existential perspective, materializes itself as a science, through NP, based on the experiences lived between “patient-being” and “nursing-being”, in “being with”, which is, in reality, a type of doing and which implies the authentic presence of “nursing-being”, constituting the meaning of these experiences and the starting point for establishing an intersubjective relationship so that NP can happen. In this sense, there is a reciprocal unveiling of one to the other, establishing an I-YOU relationship in which “nursing-being” has the possibility of carrying out, through the NP, its role as an autonomous caregiver, realizing oneself as a “caregiver being” in the world of CKD and, thus, transcending towards one’s “being more”^(21-22)^.

In light of humanistic theory^(9)^, nursing does not imply a merely fortuitous encounter, but an encounter in which there is a call and a response with determined ends, being considered a special type of lived dialogue^(9)^. “Meeting” in the world of CKD is a movement that is related to a call and a response, and becoming concerned is always present in it, as the relationship between “being” and the world is essentially becoming concerned. What exists is not the object of a theoretical world, but essentially of one whose becoming concerned imposes its presence^(4,9,21)^. Becoming concerned is related to empathy, which is understood as a way of relating, being one of the essences (values and beliefs) present in NP of humanistic nursing^(9,15,23)^.

Furthermore, finding oneself requires a space, a place, in which the context of care takes place, which in this existential sense refers not only to the physical space in which it occurs, but, above all, to the interrelationships that are built over time and that reflect the way in which beings continue to exist in this world of CKD. Therefore, it allows the redefinition of this environment as a space for social events, between family members, where it is possible to coexist and relate to other beings, creating a space for coexistence^(9,15,21)^.

The world of CKD is full of challenges, which require beings in this world to seek strategies to “continue being” and, thus, project themselves towards their “being-more”. It is clear from the lived experiences of “patient-being” that transcending CKD involves accepting it in all its complexity and finding possibilities to live better with it, which does not always happen smoothly. Even though they are aware of the need to undergo RRT, “patient-being” often tries to cling to a reality in which CKD is not present, seeking to see the treatment as something temporary^(16)^.

Moreover, during NP development, it is important that there is clear communication of CKD diagnosis to “patient-being”. It is known that sufficient knowledge related to the disease can support patient-being adherence to treatment and improve self-management skills, contributing to a better adaptation to RRT, as they feel a little more autonomous, reinforcing the importance of practicing health education through “nursing-being”^(21)^.

However, even receiving guidance, beings-in-the-world with CKD experience unpredictability, in addition to the multiple symptoms that CKD brings with it^(24)^, which ends up generating a feeling of anxieties that existentially is understood as a phenomenon revealing the structure of “being”, which expresses its deepest feeling^(4)^. In the world of CKD, the dynamic nature of anxieties has often been described as experiencing a “rollercoaster” of emotions so that the emotional state oscillated between helplessness and being more in control of one’s feelings, states related to the uncertainties that CKD and RRT themselves cause^(18,25)^.

Amidst the complexities of CKD and the anxieties it causes, beings-in-the-world of CKD need to be constantly seeking to motivate themselves to continue existing. It is understood, existentially, that motivating refers to the possibility of transcending, which means that, to project itself into the world, “being” needs to find a meaning or a reason to continue “being being”^(4)^. The world of CKD, due to its severity, demands from the beings of this world a permanent struggle to remain motivated, which is not always easy.

Therefore, the importance of improving the motivation of “patient-being” during NP through information and reinforcing their behavioral skills to facilitate self-care is highlighted^(20)^. Moreover, “nursing-being” understands that motivating “patient-being” is part of their responsibilities in care meetings, which requires an empathetic look to understand how to motivate them, but also internal motivation on their part so that they can develop strategies to combat CKD^(24)^.

It is known that the perception of CKD symptoms is a process of knowing and assessing bodily sensations that is influenced by several internal factors (e.g., personal belief, affection) and external factors (e.g., social function factors, information). Generally, “patient-being” in RRT has a mistaken understanding, with a pessimistic view of treatment, associating it with imminent death, resulting in higher levels of depressive symptoms^(24)^, reinforcing a feeling of dependence on the family. However, as time passes, as this world of CKD is revealed, “patient-being” begins to allow itself to glimpse other possibilities for continuing to exist, such as undergoing transplantation, which is seen as a hope of regaining their autonomy and freedom to make their choices. Hope enables them to endure the intensive, complex, and transformative nature of RRT^(18,26)^.

Thus, the importance of beings-in-the-world of CKD to coexist and share their anxieties and perspectives in relation to the development of strategies to face the challenges imposed by CKD and the respective RRT becomes evident, what happens over time as treatment is incorporated into daily routine, with the help of new technologies and more humanized care practices, such as NP development based on a framework that values the existential dimension of being.

### Study limitations

A limitation of this study is understood to be the fact that the sample is composed of “beings” restricted to a specific regional scenario of the country, limiting the generalization of this theory to other populations and contexts as well as the methodological design itself that does not support external validity. Thus, it is recommended that further studies be carried out in other locations, with the aim of completing its validation and, thus, if necessary, refining its concepts and the relationships established between them, in order to elevate it to a predictive and prescriptive level.

### Contributions to nursing and health

MRT of the existential dimension of being-in-the-world of CKD can be a useful tool in the clinical practice of caring for patients with renal failure, aiming to help “nursing-being” and other nephrology professionals to have the knowledge and critical thinking in decision-making to help “patient-being” to adhere to treatment and better face this moment.

This MRT is a soft-hard technology that guides NP performance to understand the qualitative evidence present in this care context and the impact it has on the life of “patient-being”, which must be observed and recorded during clinical practice in the different RRT.

## FINAL CONSIDERATIONS

This work enabled MRT development, through inductive-deductive means, based on data collected from care practice. This theory describes and explains how to recognize the qualitative evidence inherent to the existential dimension of being-in-the-world of CKD - “nursing-being” and “patient-being”.

The MRT of the existential dimension of CKD’s being-in-the-world guides clinical practice towards valuing the existential dimension of being, through bond establishment, adequate reception, contributing to “patient-being” adhering to treatment and developing coping strategies that enable them to transcend the challenges of CKD, impacting a better quality of life for this patient.

The development of this theory based on a humanistic framework, which seeks to value the uniqueness of each being in the context of CKD, contributes to nursing consolidation as an art and science, because it is born from care practice and research, in addition to rescuing what differentiates it within health disciplines, which is care par excellence.
