# Supplementary material for: Middle-range theory of the existential dimension of being-in-the-world of chronic kidney disease: Grounded Theory
Source: Rev Bras Enferm. 2024 Sep 20;77(4):e20230152. doi: 10.1590/0034-7167-2023-0152 (PMC11419687; doi:10.1590/0034-7167-2023-0152)
Supplement: Translation

# Translation: Teoria de médio alcance da dimensão existencial do ser-no-mundo da doença renal crônica: Teoria Fundamentada

**DOI:** 10.1590/0034-7167-2023-0152pt

**Authors:** Carolina Giordani da Silva, Maria da Graça Oliveira Crossetti, Maravilla Giménez Fernández, Janaína dos Santos Prates

**Affiliations:** I Universidade Federal do Mato Grosso. Cuiabá, Mato Grosso, Brasil; II Universidade Federal do Rio Grande do Sul. Porto Alegre, Rio Grande do Sul, Brasil; III Universidad Católica San Antonio de Murcia. Murcia, Murcia, Espanha

**Keywords:** Teorias de Enfermagem, Existencialismo, Enfermagem, Teoria Fundamentada, Doença Renal Crônica

## Abstract

**Objetivos:**

desenvolver uma teoria de médio alcance da dimensão existencial do ser-no-mundo da doença renal crônica à luz da teoria humanística de enfermagem.

**Métodos:**

estudo exploratório-descritivo, qualitativo, do tipo Teoria Fundamentada em Dados, cuja questão norteadora foi: que relações teóricas podem ser estabelecidas entre a prática clínica realizada no contexto das terapias renais substitutivas e os conceitos da teoria humanística de enfermagem? Os dados foram coletados por entrevista semiestruturada com equipe de enfermagem e pacientes renais crônicos.

**Resultados:**

elaborou-se uma teoria que descreve e explica a dimensão existencial do ser-no-mundo da doença renal crônica, identificando as evidências qualitativas presentes nesse contexto.

**Considerações Finais:**

a teoria contribui para qualificação da prática clínica da enfermagem no contexto da nefrologia, consolidando a enfermagem enquanto arte e ciência, porque nasce da prática assistencial e da pesquisa, além de resgatar o que lhe diferencia dentro das disciplinas da saúde, que é o cuidado por excelência.

## INTRODUÇÃO

A doença renal crônica (DRC) é caracterizada por anormalidades da estrutura ou da função dos rins presentes por mais de três meses, além da perda progressiva e irreversível da função renal, sendo parte do grupo de doenças crônicas não transmissíveis, que, no estágio mais avançado, o estágio 5, dialítico, representa o grau mais severo da doença, levando à necessidade de Terapia Renal Substitutiva (TRS) para a manutenção da vida^(1-2)^.

No Brasil, conforme o último censo publicado em 2023 pela Sociedade Brasileira de Nefrologia, o número total estimado de pessoas com DRC em estágio 5 dialítico chegava a 148.363, com aumento médio de 5.468 pacientes (3,5%) em relação ao último ano^(3)^. A TRS mais predominante para DRC é a hemodiálise, com prevalência de até 95,3% dos pacientes, seguida de diálise peritoneal e transplante renal. As TRS, embora sejam a única possibilidade de sobrevivência para os pacientes, traz com elas uma série de mudanças inerentes à dimensão física, mas também à sua condição existencial, relacionada à incerteza do futuro, à proximidade com a morte e ao medo desse novo mundo que se apresenta^(2)^.

A condição existencial refere-se ao homem como ser singular, que se mostra na cotidianidade com formas de expressão que podem ser autênticas, próprias e singulares, ou inautênticas, impróprias e impessoais. Sob a ótica existencial, o ser humano possui capacidade de ver, significar e apropriar-se das coisas do mundo a partir de sua própria perspectiva^(4)^.

No contexto da DRC, essa condição existencial está relacionada aos sentimentos expressos pelos pacientes, reconhecidos como evidências qualitativas no contexto de cuidado e que exige da equipe de enfermagem um olhar que contemple todas essas necessidades, físicas e existenciais, para realizar uma assistência de qualidade, eficaz e segura, minimizando o risco para o paciente e o profissional^(5)^.

Nesse sentido, acredita-se que, por meio de referenciais teóricos que orientem os enfermeiros a identificar esses sentimentos na realização do Processo de Enfermagem (PE), esses possam ser contemplados no planejamento do cuidado, por meio de diagnósticos e cuidados de enfermagem que reflitam a dimensão existencial do indivíduo, tornando-o singular e completo, na medida em que o ser humano é visto não somente em sua dimensão biológica, mas também nas dimensões existencial, emocional, espiritual e social, contribuindo na adesão e aceitação do paciente ao tratamento proposto.

As teorias de enfermagem contribuem na organização do cuidado, na medida que agem como referencial ou estrutura organizadora, funcionando como uma representação simbólica de aspectos da realidade para orientar a prática profissional. Têm o objetivo de descrever, explicar, prever ou prescrever condições ou relações de fenômenos, orientando a aplicação do PE em diferentes contextos^(6)^.

Entre os tipos de teoria, destaca-se a teoria de médio alcance (TMA), definida como um conjunto de ideias relacionadas que estão focalizadas em uma dimensão específica de um fenômeno, incluindo um número restrito de conceitos e proposições, descritos em um nível concreto, que estão diretamente ligadas à pesquisa e à prática, as quais geram hipóteses que podem ser testadas por meio de pesquisa empírica. A TMA para enfermagem é classificada, de acordo com sua finalidade, em descritiva, explicativa e preditiva^(7)^.

Sob essa ótica, infere-se que o desenvolvimento de uma TMA específica para a enfermagem a partir de uma grande teoria^(7-8)^, como a teoria humanística de enfermagem de Paterson e Zderad^(9)^, possa subsidiar o enfermeiro a identificar as evidências qualitativas do paciente em TRS, cuja condição existencial necessita ser avaliada no processo de cuidado da enfermagem, já que a não compreensão do processo de saúde-doença, bem como a não aceitação da sua condição, impacta negativamente o tratamento do paciente. Acredita-se que a teoria humanística^(9)^, quando propõe concretamente que as enfermeiras abordem a enfermagem consciente e deliberadamente como uma experiência existencial, venha ao encontro desse modo de olhar o “ser humano” em sua singularidade, suprindo essa lacuna na prática clínica e melhorando a qualidade do cuidado prestada a esses pacientes em TRS, já que essa população vem crescendo ano a ano^(3)^.

Nesse sentido, emergiu neste estudo a seguinte questão: que relações teóricas podem ser estabelecidas entre a prática clínica realizada no contexto da TRS e os conceitos do referencial teórico de Paterson e Zderad^(9-10)^ para a constituição de uma TMA?

## OBJETIVOS

Descrever o desenvolvimento de uma TMA da dimensão existencial do ser-no-mundo da DRC com base no referencial da teoria humanística de enfermagem de Paterson e Zderad^(9)^ com aporte da Teoria Fundamentada em Dados (TFD)^(11)^.

## MÉTODOS

### Aspectos éticos

O estudo foi conduzido de acordo com as diretrizes de ética nacionais e internacionais, e foi aprovado, primeiramente, pelo Comitê de Ética em Pesquisa da Universidade Federal do Rio Grande do Sul, e após, pelo Comitê de Ética do Hospital de Clínicas de Porto Alegre.

### Referencial teórico

A teoria da prática da enfermagem humanística propõe que enfermeiros abordem a enfermagem com deliberada consciência de que se trata de uma experiência existencial. Assim, ao refletir sobre seus conceitos, podem descrever fenomenologicamente os estímulos que recebem, suas respostas e o que chegam a conhecer por meio da sua presença na situação de cuidado ao próximo^(9)^.

A enfermagem humanística é uma experiência vivida entre seres humanos; portanto, é mais que uma relação unilateral sujeito-objeto, tecnicamente competente e caridosa, guiada em benefício de outro. É uma relação transacional que se responsabiliza por investigar, cuja expressão demanda a conceituação embasada na consciência existencial que o enfermeiro tem de seu ser e do outro^(9)^.

### Tipo de estudo

Trata-se de estudo de natureza exploratória-descritiva, com abordagem qualitativa^(11)^, com o referencial metodológico da TFD, na vertente construtivista de Charmaz^(12)^, seguindo o instrumento do EQUATOR - *Standards for reporting qualitative research* ^(13)^.

### Procedimentos metodológicos

O estudo aconteceu em um hospital universitário do Rio Grande do Sul, Brasil, no serviço de nefrologia, o qual atende pacientes renais agudos ou crônicos com necessidade de TRS.

A população do estudo foi composta por enfermeiros e técnicos de enfermagem que atuam na unidade de nefrologia e pacientes que estavam em alguma modalidade de TRS. Os participantes foram definidos por conveniência, por meio de convite para participar do estudo.

A amostragem inicial partiu de cinco enfermeiros, três técnicos de enfermagem e nove pacientes. Entretanto, de acordo com a amostragem teórica, em atenção ao referencial metodológico^(12)^, conforme os dados foram sendo coletados e os conceitos teóricos ganhavam densidade, a partir da comparação constante, uso de memos e pensamento indutivo-dedutivo, as hipóteses foram surgindo, orientando a ampliação dos participantes, resultando em uma amostra final composta por sete enfermeiros, três técnicos de enfermagem e dez pacientes. Esse quantitativo foi definido a partir do momento em que as informações dos participantes não forneciam dados relevantes em direção a novas categorizações e formulação de novos conceitos teóricos.

Os critérios de inclusão foram enfermeiros e técnicos de enfermagem com, no mínimo, um ano de atuação na instituição campo do estudo, presentes no período de coleta de dados e pacientes que estavam em algum tipo de TRS em condições de responder à entrevista. Os critérios de exclusão foram enfermeiros e técnicos de enfermagem que não estavam atuando na assistência no período da pesquisa, bem como pacientes que tivessem déficit cognitivo ou neurológico que não os permitissem responder à pesquisa e apresentassem dificuldades com uso de tecnologias, tais como aplicativos de celular e/ou computador para entrevistas virtuais.

A coleta e análise dos dados ocorreu de janeiro de 2020 a janeiro de 2021, de forma concomitante, enfatizando a elaboração das análises da ação e do processo. Dessa forma, simultâneas ajudam a prosseguir em busca dessa ênfase na medida em que adapta-se à coleta de dados, para informar as análises emergentes^(12)^. Foram realizadas entrevistas semiestruturadas, virtualmente, por meio do aplicativo Zoom, após a assinatura do Termo de Consentimento Livre e Esclarecido (TCLE), obtido de todos os indivíduos no estudo por meio escrito.

As entrevistas tiveram entre 25 e 120 minutos de duração, sendo todas gravadas. Em atenção ao referencial metodológico adotado neste estudo^(12)^, foram construídos os memorandos, que são similares aos diários de campo, em que o pesquisador, que é enfermeiro especialista em nefrologia e com sólida experiência em TRS, registrava seus pensamentos em relação ao tema, comparações e conexões realizadas, e indicava as questões e as direções a serem seguidas.

Para análise dos dados, foi utilizado o recurso do *software* NVivo 12 para realizar as codificações^(12)^. Desse modo, foram realizados os três tipos de codificação: inicial, fragmentando os dados e criando códigos “*in vivo*” proveniente das falas; focalizada, integrando, organizando e sintetizando os dados por similitude em direção às categorias e axial, relacionando as categorias e especificando suas propriedade em direção à definição dos conceitos, pressupostos, enunciados relacionais, indicadores empíricos e estrutura; e contextualização da TMA da dimensão existencial do ser-no-mundo da DRC, com base nos pressupostos e conceitos da teoria humanística de enfermagem de Paterson e Zderad^(9)^, tais como ser, encontro, preocupação, empatia, “ser-mais”, pertencimento, entre outros, os quais orientaram a construção da TMA, aproximando conceitos e estabelecendo a relação entre eles^(9)^.

## RESULTADOS

Os participantes representantes dos profissionais de enfermagem foram de predominância do sexo feminino, sendo apenas dois profissionais, um enfermeiro e um técnico de enfermagem do sexo masculino. Em relação aos enfermeiros, participaram dois enfermeiros da hemodiálise, dois da diálise peritoneal e três do transplante renal. Os técnicos de enfermagem atuavam todos na TRS de hemodiálise, com tempo de experiência que variou entre cinco e 25 anos de atuação nas TRS.

Quanto aos participantes pacientes, houve a predominância do gênero feminino, sendo apenas três pacientes do sexo masculino, com idade entre 26 e 66 anos, predominando cinco pacientes com idade entre 32 e 39 anos, demonstrando o acometimento da DRC em adultos jovens. A TRS prevalente foi a hemodiálise, realizada por quatro pacientes, seguida por três da diálise peritoneal e três transplantados renais. Entretanto, ressalta-se que, mesmo os pacientes que, no momento da entrevista, faziam outra modalidade de TRS, todos, em algum momento, passaram pela hemodiálise.

Em relação à teoria, essa apresenta vários componentes, como a finalidade, os conceitos, as afirmações, a estrutura conceitual, os pressupostos e as proposições. Esclarece-se que a expressão “no mundo da DRC”, utilizada nesta teoria, tem um cunho filosófico, referindo-se ao ambiente, espaço físico em que as TRS ocorrem e que o “ser-enfermagem” e o “ser-paciente”, que é o “ser-no-mundo com a DRC”, coabitam.

A TMA da dimensão existencial do ser-no-mundo da DRC em sua finalidade é descritiva e explicativa; portanto, propõe-se a orientar o “ser enfermagem”, a identificar a dimensão existencial no PE realizado ao “ser paciente” por meio do reconhecimento das evidências qualitativas presentes neste contexto, proporcionando um cuidado mais humanizado, valorizando a singularidade dos seres-no-mundo com DRC nas diferentes TRS, contribuindo para que esses sintam-se pertencentes a este mundo e possam, assim, desenvolver seu “ser-mais” ou seu maior potencial^(10)^.

No Quadro 1, apresentam-se os conceitos e pressupostos que estruturam a teoria, a partir das categorias e subcategorias que emergiram na análise dos dados. Ressalta-se que os elementos estruturantes são informações contidas em quantidade determinada de dados, permitindo uma compreensão mais abrangente do fenômeno estudado^(14)^, resultantes do processo de codificação proposto pela metodologia adotada neste estudo.

**Quadro 1:** Componentes da teoria proposta

Categoria	Subcategoria	Elementos estruturantes	Componente da teoria
Desvelando o ser-no-mundo da DRC	Ser paciente	Ser-sendo no mundo com DRC	Conceito
		Perdendo a autonomia pelas limitações	
		Acostumado a ser cuidado	
		Transcendendo a DRC	
	Ser enfermagem	Desvelando possibilidades	Conceito
		Sendo mediadora	
		Estando presente	Pressuposto
	Encontrando-se	Estabelecendo vínculos	Pressuposto
		Tornando-se uma família	
	Preocupando-se	Sendo empático	Pressuposto
Contexto de cuidado	Ambiente	Adaptando a realidade	Conceito
		Estabelecendo um espaço de convivência	
	Temporalidade	Tornando-se uma rotina	Conceito
		Passando o tempo	Pressuposto
Transcendendo o mundo da DRC	Aceitando a DRC	Renunciando suas possibilidades	Conceito
		Projetando-se no mundo com DRC	
	Projetando possibilidades para transcender	Estabelecendo estratégias de enfrentamento	Conceito
	Angústias do ser-no-mundo da DRC	Reconhecendo as incertezas	Pressuposto
	Motivando para transcender		Pressuposto

*DRC - doença renal crônica.*

A seguir, apresenta-se cada categoria com seus componentes.

### Categoria - Desvelando o ser-no-mundo da doença renal crônica

Esta categoria possibilitou reconhecer “ser paciente” e “ser-no-mundo com DRC” complexo, com muitas incertezas, limitação, diante de uma situação que, geralmente, se apresenta de forma súbita, e o “ser enfermagem”, constituído por enfermeiros e técnicos de enfermagem, que tem autonomia, que se preocupa com o “ser paciente” e que, com empatia e presença, a cada encontro de cuidado, busca, por meio do conhecimento e criatividade, um cuidado de excelência ao “ser paciente”, em que ambos podem projetar-se em direção ao seu “ser-mais”.

*O paciente renal crônico tem características muito peculiares que vão desde as diferentes doenças de base que podem ter, HAS, DM, doenças familiares, como rins policísticos,* [...]. *Eu enxergo ele como um paciente com várias limitações e que vai ficar um período longo em tratamento.* (ENF2)

*Eu não levo uma vida normal,* [...]. *A gente tenta, mas a gente não leva. Trancar a faculdade, mudar o modo de me alimentar, de viver, ter que pensar mais no que eu estou comendo, no que eu estou bebendo. Perder minha liberdade.* (P9)

*Depois do transplante, eu só pensava que eu estava livre das máquinas, eu não iria sofrer mais.* (P8)

### Categoria - Contexto de cuidado

É compreendido como um ambiente que é um espaço vivo, dinâmico, de inter-relações entre os seres-no-mundo da DRC que, na temporalidade de cada um, compartilham experiências, em busca de se adaptar à realidade para melhor estar neste mundo, o que ocorre com o passar do tempo. Neste cenário, o cuidado acontece quando se estabelece um espaço de convívio social que permite que os seres-no-mundo com DRC busquem o pertencimento a esse ambiente.

*Eu tive que me readaptar a essa nova vida* [...] *a parte ruim da diálise peritoneal é essa, tu ficas só, na outra, tu tinhas amizade, conversava, mas eu não tinha acompanhamento médico. Nessa eu tenho, então tudo tem prós e contras.* (P9)

*O ambiente é agradável porque não é um ambiente assim com um silêncio absoluto. Os técnicos, as pessoas brincam entre si, tudo dentro de um volume. Existe vida dentro da sala, não é uma coisa morta.* [...]*, funciona uma vida normal ali dentro.* [...] *no salão* [...] *eu vejo vida, vejo movimento.* (P6)

*Acho que, para alguns pacientes, é um evento social. Eu tenho pacientes que transplantaram e dizem que sentem falta é da convivência que tinham na hemodiálise.* (ENF6)

### Categoria - Transcendendo o mundo da doença renal crônica

Transcender o mundo da DRC requer do “ser paciente” aceitação da DRC com toda sua complexidade e, assim, ao encontrar-se com o “ser enfermagem”, juntos compartilhar suas angústias, buscando motivar-se para seguir sendo e, assim, projetar possibilidades para alcançar seu melhor potencial, ou seja, seu “ser-mais”.

*Quando eu comecei a fazer o tratamento, eu fiquei um bom tempo sem entender o que estava acontecendo.* [...] *não entendia muito bem o que era problema renal, então tinha muita incerteza* [...] *a gente não sabe como é, isso dá uma angústia.* (P10)

*Eu acho que, sim, realmente motivação é um papel do enfermeiro, acho que dentro da diálise bastante também. Dá uma conversada e realmente já motiva o paciente a querer seguir bem; dá um elogio para o paciente, eles já se sentem melhores, mais motivados, queridos e úteis.* (ENF7)

*Olha, guria, o transplante me deu uma nova vida, ia melhorar a minha vida pra melhor* [risos]. *Eu só peguei, acho que me arrumei e saí. Eu dizia: “Graças a Deus, vou sair dessas máquinas, dá um alívio”.* (P8)

Esses conceitos e pressupostos desvelados direcionam o ser-no-mundo da DRC a dois propósitos, pertencimento e “ser-mais”, conforme é possível visualizar no diagrama do Modelo da TMA da dimensão existencial do ser-no-mundo da DRC (Figura 1).

**Figura 1:** Modelo da teoria de médio alcance da dimensão existencial do ser-no-mundo da doença renal crônica

Após a identificação dos principais conceitos, foram elaboradas as afirmativas não relacionais, as quais correspondem à definição teórica ou operacional de cada um, conforme descrito a seguir:

“Ser paciente” - “ser-no-mundo com DRC”, complexo e que vivencia a angústia deste mundo diante de tantas incertezas. Apresenta medo, tristeza, desesperança, raiva, luto e sofrimento pela perda da autonomia e insegurança por viver constantemente a proximidade com a morte. Contudo, na relação com outros seres, percebe possibilidades para seguir sendo, o que lhes dá esperança e alegria em alcançar o seu “ser-mais”^(10)^.

“Ser enfermagem” - “ser-no-mundo da DRC” que cuida por meio da presença autêntica, movido pela preocupação em resposta a um chamado do “ser paciente”. Apresenta muitas especificidades, como autonomia, conhecimento específico para criar possibilidades diante dos desafios deste mundo, criatividade e empatia para perceber e estabelecer vínculo com os outros seres neste contexto^(10)^.

“Ambiente” - espaço físico, o mundo da DRC, o contexto de cuidado que diz respeito não somente ao espaço físico, mas, sobretudo, às inter-relações que nele são construídas com o passar do tempo e que refletem a maneira com que os seres seguem existindo no mundo. É um tipo de espaço vazio, personalizado, ao qual se sente pertencimento^(10)^.

“Temporalidade” - é o transcorrer do tempo, entendendo que existe uma experiência do tempo interno do ser, onde reside a autoconsciência, e do tempo externo, inerente às coisas do mundo, e que é na experiência da temporalidade e da existência resolvida que se radica o cuidado, que permite que o ser desenvolva um sentimento de pertencimento^(10)^.

“Aceitando a DRC” - condição resultante em que o “ser paciente” compreende que está doente, mas que não está só, que pode ter uma rede de apoio para lhe ajudar e que existe possibilidades de seguir se projetando no mundo em direção ao seu “ser-mais” por meio de uma TRS^(10)^.

“Projetando possibilidades para transcender” - capacidade de o ser-no-mundo com DRC se reinventar para atender às demandas de seu entorno imediato e encontrar um equilíbrio entre suas expectativas e a situação que está vivenciando^(10)^.

Os pressupostos são entendidos como crenças preconcebidas e aceitas como verdades nos quais a teoria se assentou, sem necessitarem de provas e, portanto, não são testados empiricamente^(8)^. A nova TMA possui seis pressupostos, que são:

“Estando presente” - condição para que o diálogo vivido da enfermagem efetivamente ocorra nos encontros entre o “ser paciente” e o “ser enfermagem”, o qual requer disponibilidade, com intenção de fazer e estar em uma relação, experienciando a situação compartilhada^(10)^.

“Encontrando-se” - reunião de seres singulares no mundo da DRC, em um movimento que exige presença e abertura para que possam se desvelar ao outro e, assim, coexistir, estabelecer vínculos e seguir sendo em direção ao seu “ser-mais”^(10)^.

“Preocupação” - modo essencial do “estar com”, que move o ser em direção ao encontro de cuidado em que há um chamado e uma resposta, permitindo uma relação transacional^(10)^.

“Passando o tempo” - condição de ser e estar no mundo da DRC, necessária para que o “ser paciente” e o “ser enfermagem”, ao se relacionarem, possam desvelar novas possibilidades para transcender e seguir coexistindo em direção ao futuro, singularmente, no seu ritmo interno, para se adaptar a essa nova realidade^(10)^.

“Angústia dos seres-no-mundo da DRC” - o sentimento mais profundo do “ser”, que é o princípio e a fonte de todos os demais. Relaciona-se à imprevisibilidade e às incertezas que a DRC e as próprias TRS provocam^(10)^.

“Motivando para transcender” - sentido ou razão para continuar “ser-sendo” e, assim, descobrir possibilidades para transcender e projetar-se no mundo^(10)^.

As afirmações relacionais ou proposições referem-se a algum tipo de relação entre dois ou mais conceitos da teoria, necessários para sua construção, sendo que podem afirmar uma associação (correlação) ou causalidade^(6)^. Conforme o método utilizado da TFD, essas proposições foram realizadas durante as codificações em direção à construção das categorias, por meio da criação de hipóteses que foram direcionando a amostragem teórica e a modulação da teoria. Nesse sentido, apresentam-se as proposições identificadas na nova TMA:

“Ser paciente” e “ser enfermagem” influenciam mutuamente o encontro de cuidado, na medida em que o “ser enfermagem” vai em direção ao “ser paciente” movido pela “preocupação”. Nesse sentido, “encontrando-se” depende da disposição do “ser paciente” em seguir sendo nesse mundo e, assim, transcender a DRC, e do “ser enfermagem” estar presente nesse encontro, para desvelar possibilidades para cuidar do “ser paciente”.

O “ambiente” influencia os “encontros”, na medida em que pode possibilitar ou não um espaço de convivência que interfere na aceitação da DRC e no sentimento de pertencimento pelo “ser paciente” nesse mundo em que, também, a “temporalidade” vai influenciar, pois a adaptação à TRS é um processo individual. Portanto, “encontrando-se” modula o estabelecimento de vínculos entre “ser paciente” e “ser enfermagem” em direção a tornarem-se uma família, em que o “passando o tempo” vai influenciando e, também, modulando esses encontros, em direção a cada “ser” alcançar seu “ser-mais”.

“Preocupando-se” influencia e modula a resposta do “ser enfermagem” ao “ser paciente” que busca, por meio da empatia e da mediação, influenciar a “aceitação da DRC” pelo “ser paciente”, “motivando-o” a, juntos, encontrarem “estratégias de enfrentamento” da DRC. Nesse sentido, “motivando” influencia e modula “ser paciente”, “ser enfermagem”, bem como “projetando possibilidades no mundo da DRC”, sendo um condicionante para “transcender o mundo da DRC”.

“Transcendendo o mundo da DRC” sofre interferência das “angústias dos seres-no-mundo da DRC”, na medida em que essa, muitas vezes, dificulta o “ser paciente” em aderir à TRS e o “ser enfermagem” a desvelar possibilidades para cuidar do “ser paciente”, o que também pode impactar o estabelecimento de vínculos no “encontrando-se”^(10)^.

## DISCUSSÃO

Existencialmente, o “ser” é visto como uma experiência concreta e vivida em direção a “vir-a-ser” por meio de suas escolhas. As experiências vividas têm o homem como objeto de estudo visto como uma experiência, um projeto inacabado da obra, tornando-se uma realidade a ser constantemente descoberta^(9,15)^. Assim, é necessário que se reconheça cada homem como existência singular em sua situação, relacionando-se com os outros no tempo e no espaço. É através de suas relações com outros homens que o homem chega a ser e que sua individualidade singular se realiza^(9,15)^.

Nesse sentido, o “ser paciente”, ao deparar-se no mundo da DRC, encontra-se cercado de incertezas, visto que essa apresenta-se como um acontecimento súbito, inesperado, gerando sofrimento^(16)^, além de trazer consigo complicações inerentes à sua doença de base e à própria DRC, exigindo limitações que provocam sentimento de revolta, medo, tristeza, desesperança e até isolamento social.

Além disso, a hemodiálise e a diálise peritoneal acarretam ao “ser paciente” o fardo emocional de desistir de sua liberdade enquanto passam os dias conectados a uma máquina, o que também faz surgir um sentimento de perda^(17-20)^ da sua autonomia, causando alterações significativas e sofrimento, fardo esse também referido pelos pacientes transplantados quando se remeteram a tais terapias e que deixou de existir com o transplante. Portanto, apesar disso, o “ser paciente” consegue expressar gratidão pela possibilidade de receber diálise^(14,20)^, e ao longo do tratamento, ao surgir o sentimento de pertencimento, passa a ter uma perspectiva de futuro na esperança de realizar um transplante.

Nesse sentido, a enfermagem, sob um olhar existencial, concretiza-se enquanto ciência, por meio do PE, a partir das experiências vividas entre “ser paciente” e “ser enfermagem”, no “estar com”, que é, na realidade, um tipo de fazer e que implica a presença autêntica do “ser enfermagem”, constituindo o significado dessas experiências e o ponto de partida para o estabelecimento de uma relação intersubjetiva para que o PE aconteça. Nessa acepção, há um desvelar recíproco de um para o outro, estabelecendo uma relação EU-TU em que o “ser enfermagem” tem a possibilidade de realizar, por meio do PE, seu papel de cuidador com autonomia, realizando-se enquanto “ser cuidador” no mundo da DRC e, assim, transcender em direção ao seu “ser mais”^(21-22)^.

À luz da teoria humanística^(9)^, a enfermagem não implica um encontro meramente fortuito, mas um encontro em que há um chamado e uma resposta com fins determinados, sendo considerada um tipo especial de diálogo vivido^(9)^. “Encontrar-se” no mundo da DRC é um movimento que está relacionado a um chamado e a uma resposta, e nele sempre está presente a preocupação, pois a relação do “ser” para o mundo é essencialmente preocupação. O existente não é objeto de um mundo teórico, mas essencialmente daquele cuja preocupação impõe a sua presença^(4,9,21)^. A preocupação tem relação com a empatia, a qual é compreendida como uma forma de relacionar-se, sendo uma das essências (valores e crenças) presentes no PE da enfermagem humanística^(9,15,23)^.

Além disso, encontrar-se requer um espaço, um lugar, em que se dá o contexto de cuidado, que nessa acepção existencial refere-se não somente ao espaço físico em que o mesmo ocorre, mas, sobretudo, às inter-relações que nele são construídas com o passar do tempo e que refletem a maneira em que os seres seguem existindo nesse mundo da DRC. Logo, permite o ressignificado desse ambiente como um espaço de evento social, entre familiares, onde é possível coexistir e se relacionar com outros seres, perfazendo um espaço de convivência^(9,15,21)^.

O mundo da DRC é repleto de desafios, os quais exigem dos seres neste mundo que busquem estratégias para “seguir sendo” e, assim, projetarem-se em direção ao seu “ser-mais”. Percebe-se, nas experiências vividas do “ser paciente”, que transcender a DRC passa por aceitá-la com toda sua complexidade e encontrar possibilidades de viver melhor com ela, o que nem sempre ocorre tranquilamente. Mesmo cientes da necessidade de realizar uma TRS, o “ser paciente”, frequentemente, procura a se apegar a uma realidade em que a DRC não está presente, buscando ver o tratamento como algo temporário^(16)^.

Entende-se, nesse sentido, durante o desenvolvimento do PE, que é importante que haja comunicação clara do diagnóstico da DRC ao “ser paciente”. Sabe-se que o conhecimento suficiente relacionado à doença pode apoiar a adesão do “ser paciente” ao tratamento e melhorar as habilidades de autogerenciamento, contribuindo para uma melhor adaptação à TRS, já que o mesmo se sente com um pouco mais de autonomia, reforçando a importância da prática da educação em saúde pelo “ser enfermagem”^(21)^.

Entretanto, mesmo recebendo orientações, os seres-no-mundo com DRC vivenciam a imprevisibilidade, além de múltiplos sintomas que a DRC traz consigo^(24)^, o que acaba gerando um sentimento de angústia que existencialmente é compreendido como um fenômeno revelador da estrutura do “ser”, o qual expressa seu sentimento mais profundo^(4)^. No mundo da DRC, a natureza dinâmica da angústia foi frequentemente descrita como o experimentar de uma “montanha-russa” de emoções, de modo que o estado emocional oscilava entre desamparo e estar mais no controle de seus sentimentos, estados relacionados às incertezas que a DRC e as próprias TRS provocam^(18,25)^.

Em meio às complexidades da DRC e à angústia que essa causa, os seres-no-mundo da DRC necessitam estar, constantemente, buscando motivarem-se para seguir existindo. Entende-se, existencialmente, que motivar faz referência à possibilidade de transcender, o que significa que, para projetar-se no mundo, o “ser” necessita encontrar um sentido ou uma razão para continuar “ser sendo”^(4)^. O mundo da DRC, devido à sua gravidade, exige dos seres desse mundo uma luta permanente em direção a manterem-se motivados, o que nem sempre é fácil.

Destaca-se, portanto, a importância de melhorar a motivação do “ser paciente” durante o PE por meio de informações e reforçar suas habilidades comportamentais para facilitar o autocuidado^(20)^. Nessa acepção, o “ser enfermagem” entende que motivar o “ser paciente” faz parte de suas responsabilidades nos encontros de cuidado, o que requer um olhar empático para perceber como motivá-lo, mas também uma motivação interna por parte de si, para que esse desenvolva estratégias de enfrentamento à DRC^(24)^.

Sabe-se que a percepção dos sintomas da DRC é um processo de se conhecer e avaliar as sensações corporais que é influenciado por diversos fatores internos (por exemplo, crença pessoal, afeto) e externos (por exemplo, fatores de função social, informação). Geralmente, o “ser paciente” em TRS tem uma compreensão equivocada, com uma visão pessimista sobre o tratamento, associando-o à morte iminente, resultando em níveis mais elevados de sintomas depressivos^(24)^, reforçando um sentimento de dependência com a família. Contudo, com o passar do tempo, ao ir desvelando esse mundo da DRC, o “ser paciente” começa a se permitir vislumbrar outras possibilidades para seguir existindo, como realizar o transplante, o que é visto como uma esperança de retomar sua autonomia e liberdade para fazer suas escolhas. A esperança os capacita a suportar a natureza intensiva, complexa e transformadora da TRS^(18,26)^.

Assim, fica evidente a importância dos seres-no-mundo da DRC coexistirem e compartilharem suas angústias e perspectivas em relação ao desenvolvimento de estratégias para enfrentar os desafios impostos pela DRC e as respectivas TRS, o que vai ocorrendo com o passar do tempo à medida que o tratamento vai sendo incorporado à rotina diária, com auxílio de novas tecnologias e práticas de cuidado mais humanizadas, como o desenvolvimento do PE embasado em um referencial que valoriza a dimensão existencial do ser.

### Limitações do estudo

Entende-se como limitação deste estudo o fato de a amostra ser composta por “seres” restritos a um cenário regional específico do país, limitando a generalização dessa teoria a outras populações e contextos, bem como o próprio delineamento metodológico que não sustenta uma validade externa. Nesse sentido, recomenda-se que estudos posteriores sejam realizados em outros locais, com o intuito de concretizar sua validação e, assim, se necessário, refinar seus conceitos e as relações estabelecidas entre esses, de modo a elevá-la a nível preditiva e prescritiva.

### Contribuições para as áreas da enfermagem e saúde

A TMA da dimensão existencial do ser-no-mundo da DRC pode ser uma ferramenta útil na prática clínica do cuidado ao portador de insuficiência renal, visando ajudar o “ser enfermagem” e outros profissionais da área da nefrologia a ter o conhecimento e o pensamento crítico na tomada de decisão para ajudar o “ser paciente” a aderir ao tratamento e enfrentar melhor esse momento vivido.

Essa TMA é uma tecnologia leve-dura que orienta a realização do PE para compreender as evidências qualitativas presentes neste contexto de cuidado e o impacto que provocam na vida do “ser paciente”, as quais devem ser observadas e registradas durante a prática clínica nas diferentes TRS.

## CONSIDERAÇÕES FINAIS

Este trabalho possibilitou o desenvolvimento de uma TMA, por meio indutivo-dedutivo, fundamentada em dados coletados da prática assistencial. Tal teoria descreve e explica como reconhecer as evidências qualitativas inerentes à dimensão existencial do ser-no-mundo da DRC - “ser enfermagem” e “ser paciente”.

A TMA da dimensão existencial do ser-no-mundo da DRC orienta a prática clínica em direção à valorização da dimensão existencial do ser, por meio do estabelecimento de vínculos, acolhimento adequado, contribuindo para que o “ser paciente” tenha adesão ao tratamento e desenvolva estratégias de enfrentamento que o possibilite transcender os desafios da DRC, impactando numa melhor qualidade de vida para este.

O desenvolvimento dessa teoria embasada em um referencial humanista, que busca valorizar a singularidade de cada ser no contexto da DRC, contribui para a consolidação da enfermagem enquanto arte e ciência, porque nasce da prática assistencial e da pesquisa, além de resgatar o que lhe diferencia dentro das disciplinas da saúde, que é o cuidado por excelência.
